# Long and Very-Long-Chain Ceramides Correlate with A More Aggressive Behavior in Skull Base Chordoma Patients

**DOI:** 10.3390/ijms20184480

**Published:** 2019-09-11

**Authors:** Emanuele La Corte, Michele Dei Cas, Alberto Raggi, Monica Patanè, Morgan Broggi, Silvia Schiavolin, Chiara Calatozzolo, Bianca Pollo, Carlotta Pipolo, Maria Grazia Bruzzone, Giuseppe Campisi, Rita Paroni, Riccardo Ghidoni, Paolo Ferroli

**Affiliations:** 1 PhD School in Molecular and Translational Medicine, Department of Health Sciences, University of Milan, 20142 Milan, Italy; 2 Department of Neurosurgery, Fondazione IRCCS Istituto Neurologico “Carlo Besta”, 20133 Milan, Italy; 3 Clinical Biochemistry and Mass Spectrometry Laboratory, Department of Health Sciences, University of Milan, 20142 Milan, Italy; 4 Neurology, Public Health and Disability Unit, Fondazione IRCCS Istituto Neurologico “Carlo Besta”, 20133 Milan, Italy; 5 Neuropathology Unit, Fondazione IRCCS Istituto Neurologico “Carlo Besta”, 20133 Milan, Italy; 6 Otolaryngology Unit, ASST Santi Paolo e Carlo, Department of Health Sciences, University of Milan, 20142 Milan, Italy; 7 Neuroradiology Department, Fondazione IRCCS Istituto Neurologico “Carlo Besta”, 20133 Milan, Italy

**Keywords:** cancer, ceramides, chordoma, dihydroceramides, lipid targets, skull base, sphingolipids

## Abstract

Background: Skull base chordomas are rare tumors arising from notochord. Sphingolipids analysis is a promising approach in molecular oncology, and it has never been applied in chordomas. Our aim is to investigate chordoma behavior and the role of ceramides. Methods: Ceramides were extracted and evaluated by liquid chromatography and mass spectrometry in a cohort of patients with a skull base chordoma. Clinical data were also collected and correlated with ceramide levels. Linear regression and correlation analyses were conducted. Results: Analyzing the association between ceramides level and MIB-1, total ceramides and dihydroceramides showed a strong association (*r* = 0.7257 and *r* = 0.6733, respectively) with MIB-1 staining (*p* = 0.0033 and *p* = 0.0083, respectively). Among the single ceramide species, Cer C24:1 (*r* = 0.8814, *p* ≤ 0.0001), DHCer C24:1 (*r* = 0.8429, *p* = 0.0002) and DHCer C18:0 (*r* = 0.9426, *p* ≤ 0.0001) showed a significant correlation with MIB-1. Conclusion: Our lipid analysis showed ceramides to be promising tumoral biomarkers in skull base chordomas. Long- and very-long-chain ceramides, such as Cer C24:1 and DHCer C24:1, may be related to a prolonged tumor survival and aggressiveness, and the understanding of their effective biological role will hopefully shed light on the mechanisms of chordoma radio-resistance, tendency to recur, and use of agents targeting ceramide metabolism.

## 1. Introduction

Chordoma is a rare tumor that derives from notochordal cells remnants. Although it can potentially occurs at any point along the vertebral column, the sacro-coccygeal and the skull base areas represent the most common locations [1,2,3]. Gross total tumor resection and post-operative high-dose radiation therapy actually represent the best standard of care [4,5,6]. The morbidity of skull base chordomas (SBC) is largely due to their tendency to recur and locally progress, although systemic metastases have been reported in around 10% of cases [1,4]. Their local aggressive growth pattern is characterized by encasement of neurovascular structures, dural penetration, bone infiltration, and brainstem adhesions which make chordoma a malignant tumor [1,3,5,7]. Large retrospective studies highlighted a trend towards the improvement of the survival time mainly due to the advancement of technology and surgical techniques [5,8,9,10,11,12]. Although these are great improvements, chordoma still impacts patients’ quality of life with 5-year overall survival (OS) and 5-year progression free-survival (PFS) rates of 78.4% and 50.8%, respectively [4]. Previous studies have highlighted that tumor morbidity and mortality seem to be affected not only by clinical variables, such as extent of resection, tumor location and post-operative radiation therapy, but also by biological features that are still not fully understood [2,3,13,14]. To date, no chemotherapeutic agent has showed its role as first-line therapy for chordomas. Precision medicine with molecular target therapies is emerging as a promising and innovative approach to directly inhibit specific molecules and their pathways known to be involved in SBC [2,15,16,17,18,19].

Sphingolipids act as bio-active molecular mediators in different cellular functions such as stress-response and downstream-signaling pathways, tumor proliferation, and resistance to treatment [20,21]. In particular, ceramides can drive programmed cell death, such as autophagy and apoptosis, in response to several stress and stimuli such as oxidative stress, ionizing radiation and chemotherapeutic agents but also be involved into cell proliferation [22,23]. Many studies have demonstrated the ceramides involvement in many types of cancers, such as breast, prostate cancers and gliomas, but nothing is known about their possible implication in chordomas [24,25,26].

The main goal of the present work is to analyze the role of ceramides (Cer) and dihydroceramides (DHCer) as prognostic biomarkers and their correlation with clinical variables to better elucidate chordoma behavior.

## 2. Results

### 2.1. Clinical and Radiological Features

**Figure 1:**
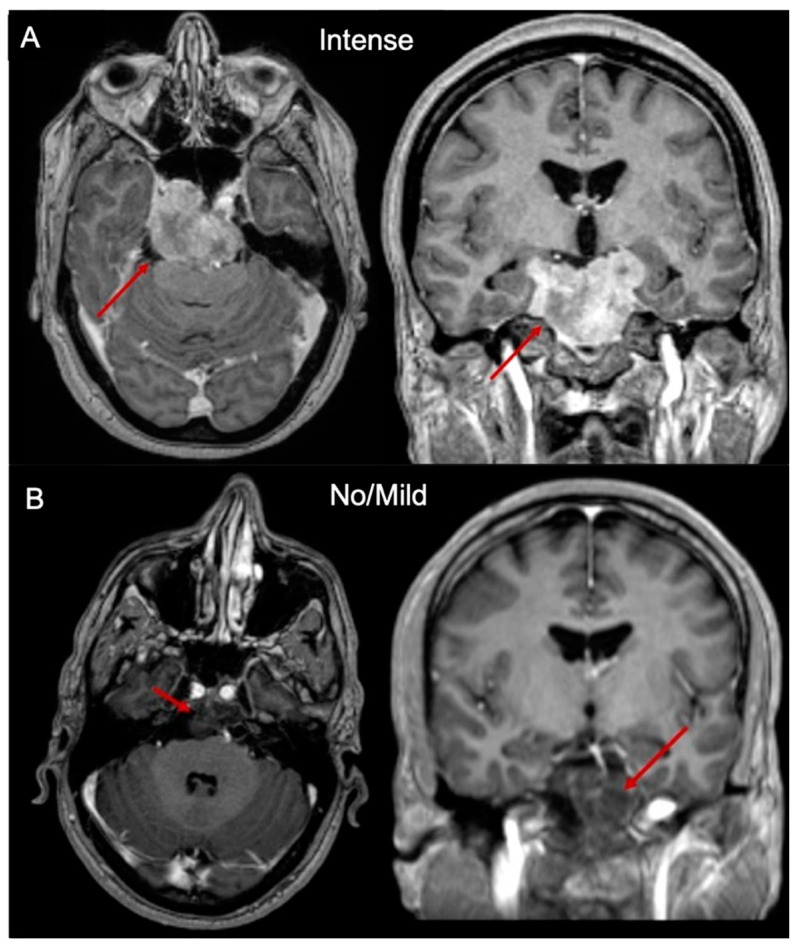
Axial and coronal T1-weighted post-contrast MR images showing SBC (red arrows) with intense (**A**) and no/mild (**B**) contrast enhancement.

Thirteen patients underwent 16 surgeries and have been included in the present study. There were seven males (53.8%) and six females (46.2%). Six tumors were primary (37.5%), eight were recurrent after radiation therapy (50%), and two were recurrent without radiation therapy (12.5%). These last two samples were gathered from the same patient who underwent a two-stage surgery; to avoid any misinterpretation of biochemical values due to surgery-induced cellular stress, only the first specimen has been included in the analysis. The mean age at the time of surgery was 53.3 ± 14.4 years (36–82). The mean follow-up period calculated from the first treatment performed at our Institution was 13.7 months (1–51). Among the recurrent chordomas, five (50%) had only one recurrence before, three (30%) had two previous recurrences, and two (20%) had three previous recurrences. The recurrent patients already treated with post-operative radiation therapy were as follows: five with carbon ions (62.5%) and three with proton beam therapy (37.5%). Preoperative radiological information was available in 14/15 of patients. One patient did not undergo contrast administration due to high serum creatinine. There were nine (64.3%) chordomas exhibiting an intense post-gadolinium MR enhancement, four (28.6%) with mild post-gadolinium MR enhancement, and one (7.1%) with no MR enhancement (Figure 1). For statistical purposes, we have identified and selected two groups: SBC exhibiting an intense post-gadolinium MR enhancement (nine chordomas) and with no/mild post-gadolinium MR enhancement (five chordomas).

### 2.2. Sphingolipids Characterization and Tumor Type

Total and single ceramides and dihydroceramides species production have been evaluated on 15 chordomas: primary chordomas (*n* = 6), recurrent without radiotherapy (*n* = 1) and recurrences after radiotherapy (*n* = 8). The mean total ceramides and dihydroceramides species in chordomas were 1216.2 ± 730.7 pmol/mg protein (522.5–2787.5) and 52.6 ± 40.6 pmol/mg protein (9.0–145.6), respectively. The mean total ceramides and dihydroceramides species in primary chordomas were 808.4 ± 451.4 pmol/mg (522.5–1760.2) and 30.7 ± 16.4 pmol/mg (17.6–62.4), respectively. The mean total ceramides and dihydroceramides species in recurrent chordomas were 1488.1 ± 763.8 pmol/mg (540.7–2787.5) and 67.2 ± 45.5 pmol/mg (9.0–145.6), respectively. Eight ceramides subspecies were analyzed and Cer C16:0 (60.8%), Cer C24:1 (17.5%), and Cer C24:0 (10.0%) were the most abundant single ceramides in SBC. In the primary chordoma group, the most abundant were Cer C16:0 (66.1%), Cer C24:1 (13.8%), and Cer C24:0 (10.1%). In the recurrent chordoma group, the most abundant were Cer C16:0 (58.9%), Cer C24:1 (18.8%), and Cer C24:0 (9.9%). Five dihydroceramides were analyzed and DHCer C16:0 (60.5%), DHCer C24:1 (24.8%), and DHCer C24:0 (11.4%) were the most abundant single ceramides in SBCs. In the primary chordoma group, the most abundant were DHCer C16:0 (70.4%), DHCer C24:1 (17.7%), and DHCer C24:0 (9.5%). In the recurrent chordoma group, the most abundant were DHCer C16:0 (57.5%), DHCer C24:1 (27.0%), and DHCer C24:0 (11.4%).

**Figure 2:**
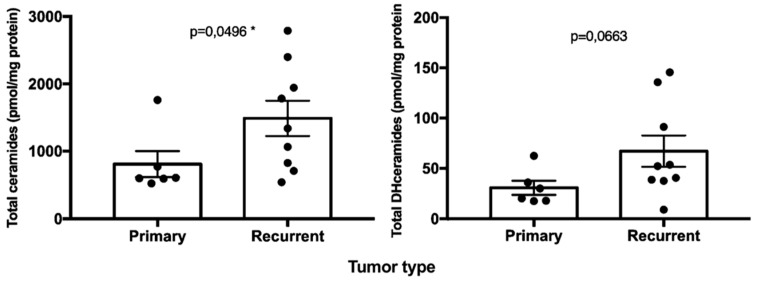
Bar graphs, with standard error mean (SEM), showing the content of total ceramides (*, left) and dihydroceramides (right) species according to tumor type (primary and recurrent chordoma groups). *, *p* ≤ 0.05.

Total ceramides species (Figure 2) were significantly higher in recurrent chordomas that underwent previous surgical resection and radiation therapy in comparison to the primary chordomas (*p* = 0.0496). When analyzing the effect of radiation therapy, the ceramides in the group of chordomas with previous radiation therapy were not significantly different from the ceramides in the group of chordomas with no previous radiation therapy (*p* = 0.0541). DHCer levels (Figure 2) were not significantly different between primary and recurrent chordomas (*p* = 0.0663) and between chordoma with previous radiation therapy and chordoma without radiation therapy (*p* = 0.1520).

**Figure 3:**
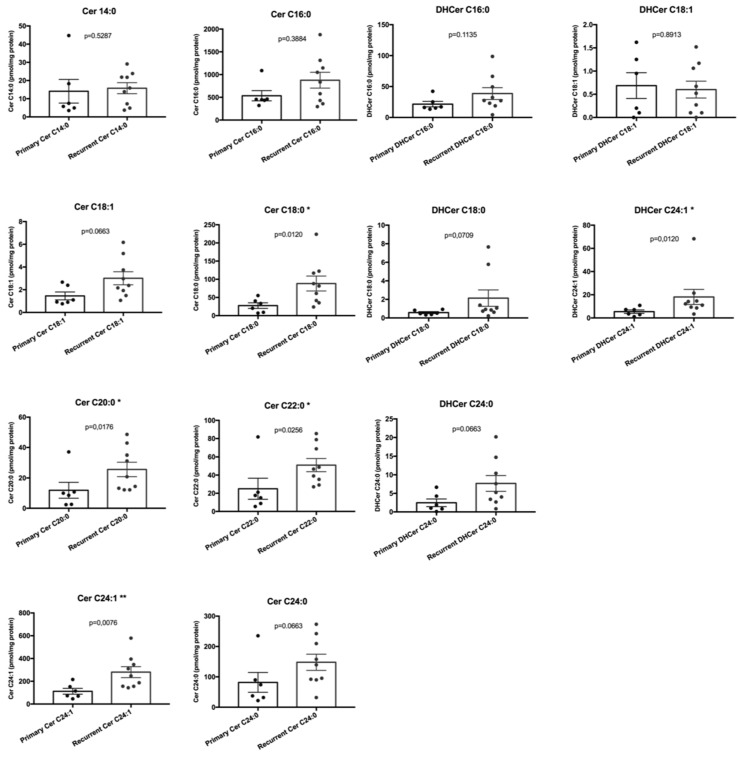
Bar graphs, with SEM, showing the content of single ceramides and dihydroceramides species according to tumor type (primary and recurrent chordoma groups). *, *p* ≤ 0.05; **, *p* ≤ 0.01.

Among the single ceramides species (Figure 3), Cer C18:0 (*p* = 0.0120), Cer C20:0 (*p* = 0.0176), Cer C22:0 (*p* = 0.0256), and Cer C24:1 (*p* = 0.0076) were significantly different between primary and recurrent SBCs, whereas Cer C14:0 (*p* = 0.5287), Cer C16:0 (*p* = 0.3884), Cer C18:1 (*p* = 0.0663), and Cer C24:0 (*p* = 0.0663) did not reach any statistical significance. Among the single DHCer species (Figure 3), DHCer C24:1 (*p* = 0,0120) was the only one significantly different between primary and recurrent SBCs, whereas DHCer C16:0 (*p* = 0.1135), DHCer C18:1 (*p* = 0.8913), DHCer C18:0 (*p* = 0.0709), and DHCer C24:0 (*p* = 0.0663) did not reach any statistical significance.

### 2.3. Sphingolipids Characterization and MR Contrast Enhancement Pattern

**Figure 4:**
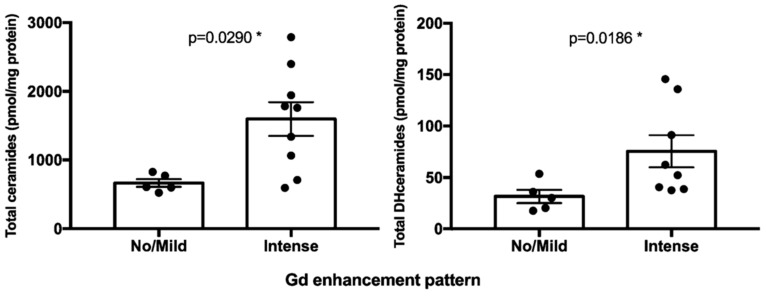
Bar graphs, with SEM, showing the content of total ceramides (*, left) and dihydroceramides (*, right) species according to MR contrast enhancement (no/mild and intense contrast enhancement chordoma groups). *, *p* ≤ 0.05.

The mean total ceramides and dihydroceramides species in the “intense enhancement” group were 1597.6 ± 737.8 pmol/mg (592.7–2787.5) and 69.1 ± 45.0 pmol/mg (17.8–145.6), respectively. The mean total ceramides and dihydroceramides species in the “no/mild enhancement” group were 664.7 ± 120.4pmol/mg (522.5–826.0) and 31.5 ± 13.6 pmol/mg (17.6–53.6), respectively. Total Cer and DHCer levels (Figure 4) were significantly higher in “intense enhancement” chordomas in comparison to the “no/mild enhancement” chordomas (*p* = 0.0290 and *p* = 0.0186, respectively).

**Figure 5:**
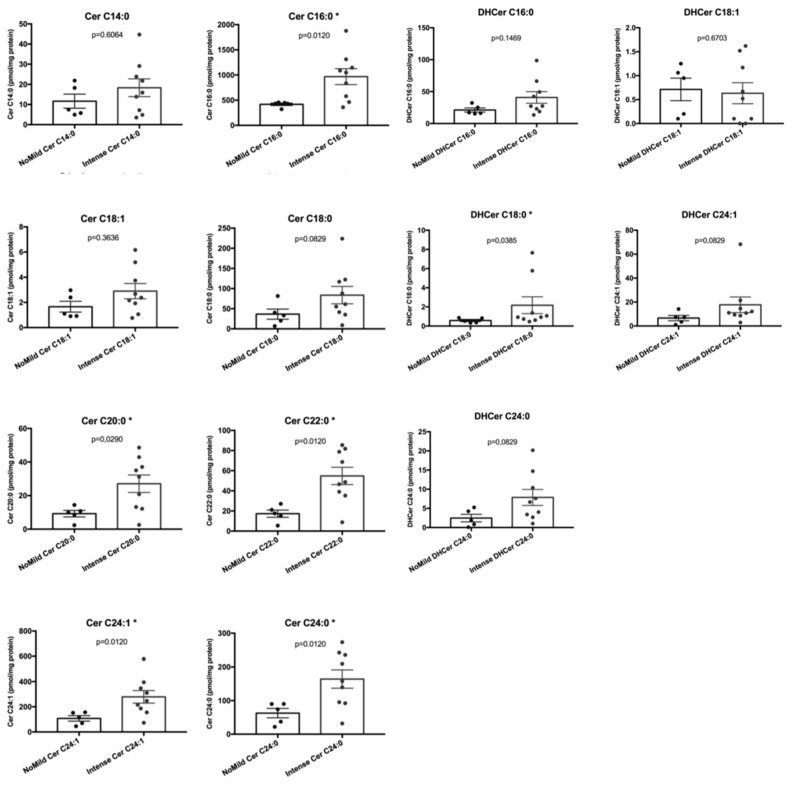
Bar graphs, with SEM, showing the content of single ceramides and dihydroceramides species according to MR contrast enhancement (no/mild and intense contrast enhancement chordoma groups). *, *p* ≤ 0.05.

Among the single ceramides species (Figure 5), Cer C16:0 (*p* = 0.0120), Cer C20:0 (*p* = 0.0290), Cer C22:0 (*p* = 0.0120), Cer C24:1 (*p* = 0.0120), and Cer C24:0 (*p* = 0.0120) were significantly different between “intense enhancement” chordomas and “no/mild enhancement” chordomas, whereas Cer C14:0 (*p* = 0.6064), Cer C18:1 (*p* = 0.3636), and Cer C18:0 (*p* = 0.0829) did not reach any statistical significance. Among the single DHCer species (Figure 5), DHCer C18:0 (*p* = 0.0385) was the only one significantly different between “intense enhancement” chordomas and “no/mild enhancement” chordomas, whereas DHCer C16:0 (*p* = 0.1469), DHCer C18:1 (*p* = 0.6703), DHCer C24:1 (*p* = 0.0829), and DHCer C24:0 (*p* = 0.0829) did not reach any statistical significance.

### 2.4. Sphingolipids Characterization and Proliferative Index

**Figure 6:**
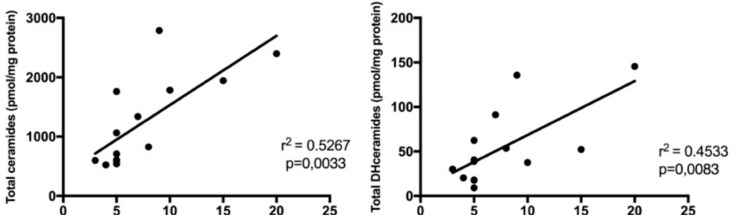
Linear regression graphs showing the correlation between the rate of MIB-1 staining and total ceramides.

Following the hypothesis of ceramides as aggressive behavior biomarkers of SBC, we, therefore, investigated the relationships between Cer and DHCer levels with the tumor proliferation rate, evaluated by MIB-1 staining on IHC slices. MIB-1 staining information was available in 14/15 of patients. Analyzing the association between Cer levels and MIB-1 within each SBC patient (Figure 6), total ceramides levels showed a strong association (*r* = 0.7257, *r*^2^ = 0.5267) with MIB-1 staining (*p* = 0.0033). Analyzing the association between DHCer levels and MIB-1 within each SBC patient (Figure 6), total DHCer levels showed also a moderate association (*r* = 0.6733, *r*^2^= 0.4533) with MIB-1 staining (*p* = 0.0083).

**Figure 7:**
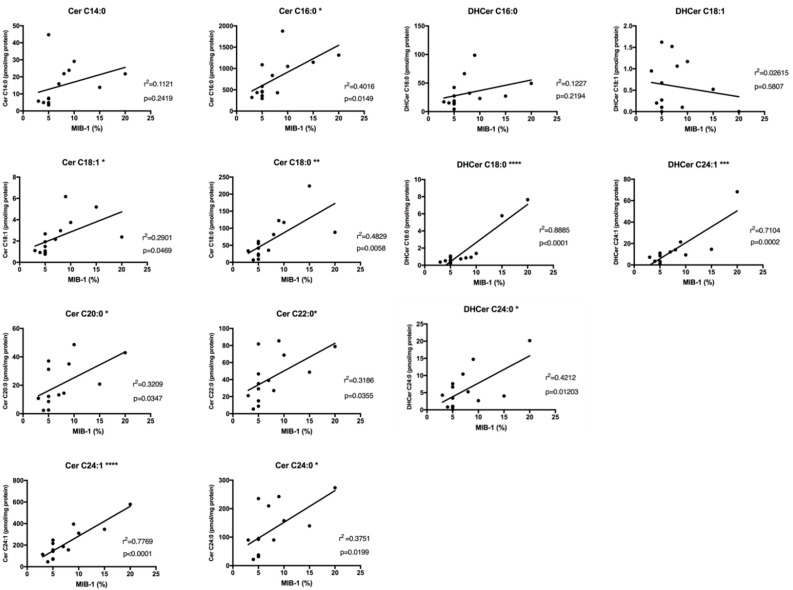
Linear regression graphs showing the correlation between the rate of MIB-1 staining and single ceramides and dihydroceramides species. *, *p* ≤ 0.05; **, *p* ≤ 0.01; ***, *p* ≤ 0.001, ****, *p* ≤ 0.0001.

In particular, we have analyzed any possible association between every single ceramide species and MIB-1 (Figure 7) and found that Cer C16:0 (*r* = 0.6338, *r*^2^ = 0.4016, *p* = 0.0149), Cer C18:1 (*r* = 0.5386, *r*^2^ = 0.2901, *p* = 0.0469), Cer C18:0 (*r* = 0.6949, *r*^2^ = 0.4829, *p* = 0.0058), Cer C20:0 (*r* = 0.5665, *r*^2^ = 0.3209, *p* = 0.0347), Cer C22:0 (*r* = 0.5645, *r*^2^ = 0.3186, *p* = 0.0355), Cer C24:1 (*r* = 0.8814, *r*^2^ = 0.7769, *p* ≤ 0.0001), and Cer C24:0 (*r* = 0.6125, *r*^2^ = 0.375, *p* = 0.0199) levels showed a significant correlation with MIB-1 staining. Moreover, we have also analyzed an association between every single dihydroceramides species and MIB-1 (Figure 7) and found that DHCer C18:0 (*r* = 0.9426, *r*^2^ = 0.8885, *p* ≤ 0.0001), DHCer C24:1 (*r* = 0.8429, *r*^2^ = 0.7104, *p* = 0.0002) and DHCer C24:0 (*r* = 0.649, *r*^2^ = 0.4212, *p* = 0.0120) levels showed a significant correlation with MIB-1 staining. The results of correlation analysis are shown in Table 1.

A regression model has been developed to evaluate the role of ceramides and dihydroceramides as independent predictors of MIB-1 (Table 2). The level of significance was set to 0.05/15 = 0.0033, according to Bonferroni’s correction. All the independent variables were entered into the equation first and each one was deleted one at a time if they did not contribute to the regression equation by the backward elimination method. A multi-collinearity test has been used to assess the Variance Inflation Factor (VIF) and tolerance. Final candidate predictive factors that well fitted the model were: Cer C24:1 (*r* = 0.824, *p* ≤ 0.001) and DHCer C18:0 (*r* = 0.748, *p* = 0.002).

## 3. Discussion

This study represents the first sphingolipid analysis in SBCs surgical specimens. Previous studies have demonstrated that a delicate equilibrium exists between long-chain (i.e. Cer C16:0, Cer C18:0, Cer C20:0) and very-long-chain (Cer C24:1, Cer C24:0) ceramides to determine their specific biological role in regulating apoptosis and proliferation pathways [23,27]. The specific ultrastructural structure of chordomas consists of many vacuoles and lipid raft-like regions of endoplasmic reticulum tightly linked with mitochondrial membranes, called MAM mitochondria-associated ER membrane [28]. These structures have been found to be involved in lipid synthesis, apoptosis and cell proliferation [28,29,30]. Based on such observations, we decided to perform a ceramide analysis in chordoma samples.

Our preliminary targeted analysis showed that recurrent skull base tumors presented a higher level of total ceramides than the primary ones (*p* ≤ 0.05). Moreover, single ceramides species have never been correlated with clinical variables and no definitive biological role could be hypothesized in chordomas. The regression and correlation analyses showed that very-long-chain ceramides (such as Cer C24:1) were significantly different between primary and recurrent chordomas (*p* = 0.0076). Also, the very-long-chain DHCer C24:1 (*p* = 0.0120) was the only species that was significantly different related to tumor type. Recurrent patients have undergone previous combined treatments such as surgery and RT and both factors may explain why ceramides are significantly different in the two groups [25,31,32,33]. Since the recurrent tumor type and previous treatments represented a significant negative prognostic factor relating to worse OS and PFS in previous studies, a raising in ceramides content in SBCs could act as a negative prognostic factor [16,34,35,36]. We showed that there is also a concomitant rise in both ceramides and dihydroceramides and such a finding could be related to an activation of the de-novo pathway, but further analyses are needed. De novo pathway represents a strong autophagy inducer [21,37,38,39]. In vitro, autophagy could trigger both a cytoprotective action promoting radio-resistance and a cytotoxic function [40,41,42,43]. Autophagy induces the activation of many cellular pathways, and damaged organelles and proteins are conveyed to degradation to maintain cellular homeostasis [42,44,45,46]. For example, ionizing radiation induces mitochondrial damage, which triggers autophagy mediated by E3-ubiquitin ligase p62 [31]. Different molecules have been developed as inhibitors of serine palmitoyltransferase (SPT), the first enzyme involved in the de-novo synthesis pathway. Some potent and selective inhibitors have been isolated from microorganisms, such as sphingofungins, lipoxamycins, viridiofungins, and myriocin (Myr) [47,48]. Several studies showed the efficacy of Myr treatment, the only commercially available product, either in in-vivo or in-vitro models and its capacity to irreversibly inhibit SPT [47,49]. Myr showed to also have a potential role in reducing tumor cell proliferation through SPT inhibition [47,50]. The involvement of de-novo ceramide synthesis has been shown in different immunometabolic diseases clusters such as obesity, diabetes and neurodegeneration, cystic fibrosis, and retinitis pigmentosa [51,52,53,54,55]. Several recent studies showed that intra-tracheal administration of Myr in CF mouse model infected with Pseudomonas aeruginosa or Aspergillus fumigatus showed an anti-inflammatory role due to the inhibition of de-novo sphingolipid synthesis [54,56,57,58]. Therefore, Myr may have a significant role in the future therapeutic innovation and application in chordoma patients where a de-novo ceramide synthesis seems to be over activated. More studies are needed to firstly confirm the deregulation of such ceramide biosynthesis pathways in chordoma and to assess the pharmacokinetics and pharmacodynamics of Myr.

**Figure 8:**
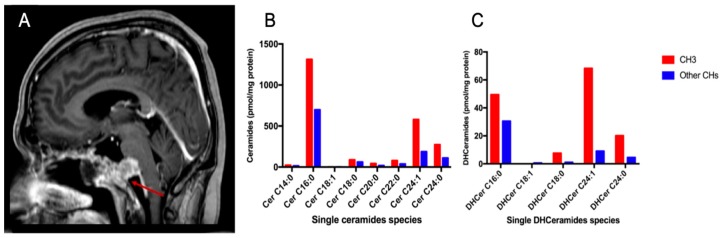
Exemplificative case of a SBC with an aggressive behavior. Sagittal (**A**) T1-weighted post-contrast MR image showing a chordoma (red arrows) with an intense contrast enhancement. Pathology showed a de-differentiated chordoma with a high rate of MIB-1 staining (20%). Sphingolipid analysis showed the highest content in ceramides (**B**) and dihydroceramides (**C**). Bar graphs showing the ceramide content of the present case (red) in comparison with the average of ceramide levels of other chordoma patients (blue).

The radiological appearance of SBC is very heterogeneous and has been scarcely described and correlated with prognosis in the literature [59,60]. The degree of contrast enhancement in chordoma is not systematically described and is not considered in the modern clinical and radiological patient management as a prognostic factor in SBC [60,61]. On the ground of previous confirmatory quantitative radiomics results, we adopted the following classification of MR enhancement pattern: intense enhancement, mild enhancement and no enhancement that revealed to be very simple to use, fast-forward, and reliable [60]. Of note, the degree of Gd enhancement in our cohort of SBC showed to strongly correlate with both OS and PFS and correlate with a more aggressive behavior [19]. It could, therefore, represent a radiological bio-marker of a more aggressive behavior (Figure 8). To further support ceramides as a bio-marker of aggressive behavior of chordomas, we tried to perform a correlation analysis in relation to the gadolinium enhancement pattern. Firstly, total ceramides and dihydroceramides levels were significantly higher in the intense gadolinium enhancement group than in the no/mild enhancement group (*p* = 0.0290). Moreover, very-long-chain single ceramides species were significantly different between the two radiological categories (*p* = 0.0120). Such results confirmed that very-long-chain ceramides and dihydroceramides may represent an intrinsic biological feature of more aggressive chordomas.

We, then, correlated the Cer and DHCer levels with tumor proliferation rate MIB-1 marker and found that they were strongly correlated (*r* = 0.7257, *p* = 0.0033 and *r* = 0.6733, *p* = 0.0083, respectively). Higher levels of sphingolipids were associated with higher MIB-1 values and, therefore, to high proliferating tumors. The single ceramides and dihydroceramides species that were more correlated to high proliferative tumors were Cer C24:1 and its respective DHCer C24:1 (*r* = 0.8814, *p* ≤ 0.0001 and *r* = 0.8429, *p* = 0.0002, respectively). Moreover, the final candidate predictive factors that well fitted the regression model were Cer C24:1 (beta = 0.348, *p* = 0.025) and DHCer C18:0 (beta = 0.663, *p* ≤ 0.001).

There is a delicate balance between different CerS isoforms and the different ceramide species production within the stress-induced response and, moreover, tumoral cells can activate pro-survival mechanisms that could relate to chordoma radio-resistance [62]. A paper showed, in an in-vitro HeLa cells model, that ceramides produced by CerS5 (mainly Cer C16:0) following radiation have a pro-apoptotic role, whereas ceramides produced by CerS2 (mainly very-long-chain, Cer C24:1 and C24:0) have a pro-survival role [62]. Interestingly, Kolb et al. also showed through a lipid-targeted analysis, that a chordoma cell line presented a massive upregulation of glucosylceramides GlyCer C24:0 and GlyCer C24:1 compared with other health and pathological cell lines and postulated that these species could be related to chordoma radio-resistance [28].

Based on our results, Cer C24:1 and DHCer C18:0 may represent aggressiveness biomarkers of SBCs. The concomitant rise of DHCer C24:1 may also be explained by an exaggerated upregulation of the de-novo pathway that, in turn, may be the inducer of cytoprotective autophagy concurring to cell survival and resistance to different treatments [19,63].

Since it is a preliminary study, numerous samples are needed to confirm and externally validate our results. Chordoma is a very rare tumor, and multi-institutional studies are, therefore, important to achieve a larger sample size. Another limitation is represented by a short follow-up of chordomas patients and, therefore, longer follow-ups are needed to further correlate ceramides levels with outcome prognostic factors such as OS and PFS. Moreover, to better asses if the de-novo pathway is actually activated, the specific involved enzyme should be ad hoc analyzed. Specifically, the expression and activity of the following enzymes could be assessed: i) de-novo sphingolipid synthesis (serine palmitoyl transferase, DH Cer synthase, Cer desaturase); ii) recycling of sphingosine to generate Cer (different Cer Synthases, from CerS1 to CerS6); iii) Cer metabolism (ceramidases, neutral and acid sphingomyelinases, glucosylceramide synthase, and glucocerebrosidase) [23,64].

## 4. Materials and Methods

### 4.1. Study Design and Population

A retrospective review of all consecutive patients diagnosed, accordingly to the 4^th^ Edition of WHO Classification of Tumours of Soft Tissue and Bone, and treated for a SBC at the Fondazione IRCCS Istituto Neurologico “Carlo Besta” between October 2015 and December 2017, was performed [1]. All the relevant clinical data were retrieved from the Institutional Registry of Neurosurgical Complications. The exclusion criteria were the following: incomplete clinical data and different location. All subjects gave their written informed consent for inclusion before they participated in the study and for using their clinical and molecular data for research purposes. All the procedures involving human subjects were done in accordance with the Helsinki Declaration of 1975. The protocol was approved by the Ethics Committee of Fondazione IRCCS Istituto Neurologico “Carlo Besta” (Project identification code: Prot. CQ-NCH). All radiological features were evaluated by two independent reviewers blinded to the clinical outcome and ceramide level. The degree (absent, mild, intense) of contrast enhancement on MR images was also evaluated [60]. Tumor specimens were prospectively collected from the operating room and verified by the pathologist. One part was fixed in Carnoy’s solution, de-hydrated in absolute ethanol and paraffin-embedded, and then sectioned at 2 μm. The second one was directly snap-frozen in liquid nitrogen and stored at −80 °C for the sphingolipid analysis. For the histological analysis, slides were re-hydrated and stained with hematoxylin-eosin (H&E) according to the standard methods. The chordoma tissue samples were also routinely investigated by immunohistochemistry (IHC) for the expression of proliferative markers MIB1/ki67 (mouse monoclonal anti-Ki67 clone MIB1 1:100, DAKO, Denmark).

### 4.2. Sphingolipids Analysis with LC-MS/MS

Ceramides and dihydroceramides species were extracted from frozen samples and assessed using liquid chromatography–mass spectrometry (LC-MS/MS) [65,66]. Tumor samples were added with 100 µL of PBS + 0.1% protease inhibitor and homogenized in TissueLyser (Qiagen, Hilden, Germany) for 3 min at 50 oscillations/s. Total protein concentration was measured by Bradford dye-binding method. Samples were added with 10 µL of IS (Cer C12 20 mM) and 850 µL of a methanol/chloroform mixture (2:1, *v/v*), then sonicated for 30 min. Afterwards, they were incubated overnight in an oscillator bath at 48 °C. Once at room temperature, samples were added with 75 µl of KOH 1 M in methanol and incubated at 37 °C for 2 h. To bring the pH to a neutral level, 75 µl of acetic acid 1 M was added in methanol. The phase was evaporated under a stream of nitrogen. The residuals were dissolved in 150 µl of methanol and then centrifuged for 10 min at 13,000 rpm. Ten microliters were directly injected in LC-MS/MS. The analytical system consisted of a HPLC coupled to a tandem mass spectrometer. The LC system was a Dionex 3000 UltiMate instrument with autosampler, binary pump and column oven (Thermo Fisher Scientific, USA). Separation was attained on a reversed-phase BEH C-8 100 × 2.1 mm × 1.7 µm analytical column preceded by a security guard cartridge. Linear gradients were obtained between eluent A (water + 2 mM ammonium formate + 0.2% formic acid) and eluent B (methanol + 1 mM ammonium formate + 0.2% formic acid). The column was equilibrated with 80% (B), increased to 90% (B) in 3 min, held for 3 min, increased to 99% (B) in 9 min, held for 3 min, back to the initial conditions in 2 min, and kept for 2 min at 80% (B). The flow rate was 0.3 mL/min, the autosampler and the column oven were kept at 15 °C and 30 °C, respectively. The tandem mass spectrometer was an AB Sciex 3200 QTRAP instrument with electrospray ionization TurboIonSpray™ source (AB Sciex S.r.l., Milano, Italy). Instruments were managed with the proprietary manufacturer’s software and according to the manufacturer’s instructions. The analytical data were processed using Analyst software (version 1.2). The ion spray voltage was set at 5.5 kV, and the source temperature was set at 300 °C. Nitrogen was used as a nebulizing gas (GS 1, 45 psi), turbo spray gas (GS 2, 50 psi) and curtain gas (25 psi). The collision-activated dissociation (CAD) was set to a low level. The dwell time was set at 0.1 s, and the MS scan was performed in positive ion modes (ESI +). MS/MS experiment were conducted using nitrogen as collision gas. Compound-dependent parameters (CE, DP) were optimized via direct infusion. Multiple reaction monitoring (MRM) mode was used. Quantitative analysis was performed interpolating each peak area of the analyte/area IS with the calibration curve of each sphingolipid. The sphingolipid amount was normalized by total protein content, expressed in milligrams, in each sample. Six-point calibration curve was evaluated by spiking increasing amounts of the analytes in water in the concentration range of 0–40 pmol/vial. Linearity was observed for each analyte in the whole range (*R*^2^ > 0.99).

### 4.3. Statistical Analysis

Descriptive statistics were used to analyze sociodemographic and clinical data. Categorical variables were described with frequencies and percentages, continuous variables with means, standard deviations, and ranges. Univariate comparisons were conducted using Chi-square and exact Fisher tests. Ceramides and dihydroceramides total and single species were subjected to multiple linear regression analysis using a Pearson correlation model with MIB-1 as a regression model target to evaluate their role as independent prognostic factors. Statistical analyses were run on SPSS v.18 (IBM Inc., Armonk, NY, USA) and GraphPad (GraphPad Software, Inc., San Diego, CA, USA).

## 5. Conclusions

Sphingolipid analysis represents a very new approach in chordomas, and future studies are needed. Our preliminary analysis showed ceramides to be promising tumoral biomarkers in chordomas. Long- and very-long-chain ceramides, such as Cer C24:1 and DHCer C18:0, may be related to a more prolonged tumor survival and aggressiveness, and the understanding of their effective biological role will hopefully shed light on the mechanisms of chordoma radio-resistance and tendency to recur. To better determine which pathway of ceramides synthesis is involved in chordoma biology, it will be useful to specifically evaluate different enzymes, such as ceramides synthases and desaturases. In-vitro studies would be useful to determine the direct relationship between hadron-therapy and ceramides formation and analysis of programmed cell-death mechanisms. In-vitro and in-vivo models could also be employed to evaluate the potential therapeutic effects of specific molecules, such as Myriocin, targeting sphingolipids’ metabolism in chordomas.

## Figures and Tables

**Table 1:** Summary of the correlation analysis between ceramides and MIB-1. *, *p* ≤ 0.05; **, *p* ≤ 0.01; ***, *p* ≤ 0.001, ****, *p* ≤ 0.0001

Variable.	*r*	*p*-value	
Cer14:0	0,3349	0,2419	ns
Cer16:0	0,6338	0,0149	*
Cer18:1	0,5386	0,0469	*
Cer18:0	0,6949	0,0058	**
Cer20:0	0,5665	0,0347	*
Cer22:0	0,5645	0,0355	*
Cer24:1	0,8814	≤ 0,0001	****
Cer24:0	0,6125	0,0199	*
DHCer16:0	0,3503	0,2194	ns
DHCer18:1	−0,1617	0,5807	ns
DHCer18:0	0,9426	≤ 0,0001	****
DHCer24:1	0,8429	0,0002	***
DHCer24:0	0,6490	0,0120	*

**Table 2:** Linear regression predicting outcome based on ceramide levels in SBC patients. *R^2^ = 0.931, Adj-*R*^2^ = 0.918; F = 74.0 (*p* ≤ 0.001).

Variable	B Value (SE)	Beta	*p*-value	Tolerance	VIF
**Constant**	2.97 (0.73)		0.002		
**Cer C24:1**	0.011 (0.004)	0.348	0.025	0.351	2.85
**DHCer C18:0**	1.40 (0.28)	0.663	≤ 0.0001	0.351	2.85
